# A simple, fast and inexpensive method for mutation scanning of CFTR gene

**DOI:** 10.1186/s12881-017-0420-9

**Published:** 2017-05-25

**Authors:** Juan Emilio Figueredo Lago, Anny Armas Cayarga, Yaimé Josefina González González, Teresa Collazo Mesa

**Affiliations:** 1 Department of Molecular Biology, Immunoassay Center (CIE) Cubanacan, Playa, Havana, Cuba; 2 National Center of Medical Genetics, Havana, Cuba

**Keywords:** Mutation scanning, CF-causing mutations, Allele-specific multiplex real-time PCR

## Abstract

**Background:**

Mutation scanning methods in Cystic Fibrosis Transmembrane Conductance Regulator (CFTR) gene may not distinguish between a Cystic Fibrosis (CF) causing mutation and a benign variant. We have developed a simple and fast method for scanning 14 selected CF-causing mutations which have high frequency in Latin America.

**Methods:**

In a group of 35 samples coming from CF patients previously characterized and using two allele-specific real-time multiplex PCRs targeting wild-type and mutant alleles respectively, we detect the presence of mutations by analyzing the Ct variation. Twenty-five samples without mutations considered non-carrier samples, were also included in this study. High Resolution Melting Analysis (HRMA) was performed to confirm the result of the scanning method and in most cases allowed the genotype determination.

**Results:**

The results validate this method for CF diagnosis. A least one CFTR gene mutation was detected in the samples of CF patients, as predicted by their ΔCt values. The ΔCt value also indicated the zygosity of the sample according to the distribution of CFTR gene mutations. In most cases, HRMA allowed the identification of the mutation(s), thereby confirming the efficiency of this scanning strategy.

**Conclusions:**

This strategy simplifies the detection of CF, reducing the analysis of 14 CF-causing mutations to two parallel reactions and making the procedure compatible with the analysis of a large number of samples. As the method is fast, inexpensive and highly reliable, it is advisable for scanning CFTR gene mutations in newborns, patients with a clinical suspicion of CF as well as in the preconception carrier screening.

## Background

Cystic fibrosis (CF) is a life-shortening hereditary disease caused by mutations in the Cystic Fibrosis Transmembrane Conductance Regulator (CFTR) gene [1]. The study of the CFTR gene represents one of the most frequent genetic analyses performed worldwide [2]. In Latin-American countries, CF and associated disorders have remained unknown entities until recently [3, 4]. Cuba, with a large genetic heterogeneity due to the multiethnic origin of its inhabitants, has reported a significant CF incidence: 1 per 9862 newborns [5].

The implementation of prenatal and preconception carrier screening using genetic analysis could detect and prevent the disease, but the availability of simple and inexpensive technology for detecting mutations on the CFTR gene is a determining factor in the CF diagnosis. Indirect detection methods such as denaturing gradient gel electrophoresis (DGGE) and High Resolution Melting Analysis (HRMA) are techniques designed to explore the gene, exon by exon, for abnormalities [6]. HRMA of PCR products is based on monitoring the fluorescence released during the melting of double-stranded DNA bound to a saturation dye [7]. Regardless their widespread use, these methods performed for CFTR gene scanning may not distinguish between a CF-causing mutation and a benign variant. This uncertainty in scanning the CFTR gene decreases the utility of these methods [8] and they are less feasible in areas in which the profile of CFTR mutations have barely been characterized.

The Amplification Refractory Mutation System (ARMS) is one of the most frequently used methods for detecting CFTR mutations [9]. Recent findings suggest the reliability of detecting individual CFTR gene mutations using the real time-ARMS PCR strategy [10, 11]. Through the combination of this last and the HRMA technique, carrier (homozygous, heterozygous) and non-carrier samples can be detected and subsequently differentiated according to their melting profile [12]. By developing a method that includes these advantages and considering frequent mutations that cause CF in Cuba, the National Health System will have a more comprehensive CF screening program and will reduce the time and cost of the analysis of patients.

## Methods

The aim of this work was to develop a cost effective method to scan 14 CF-causing mutations on the CFTR gene using a SYBR Green based real-time multiplex ARMS PCR. HRMA was also used for identifying the mutation(s) according to their amplicon melting profile. The mutations included in this method and with documented frequency in Latin America [13–15] were: R334W, I507del, F508del, 1717-1G > A, G542X, G551D, R553X, 1811 + 1,6KbA > G, 2183AA > G, 3120 + 1G > A, 3272-26A > G, R1162X, W1282X and N1303K.

We performed the mutation scanning in 60 samples, 35 of them from CF patients with a known CFTR genotype that were confirmed by the commercial CF StripAssay 4–410 panel, (ViennaLab Diagnostics, Austria) and DGGE technique. The genotypes of the samples were: R334W/*wild-type (wt)* (2*)*, I507del/*wt* (2), F508del/*wt* (3), G542X/*wt* (4), R553X/*wt* (2), 3120 + 1G > A/*wt* (2), R1162X/*wt* (2), R334W/F508del (3), I507del/F508del (4), F508del/F508del (5), 1717-1G > A/F508del (1), N1303K/F508del (1), I507del/2183AA > G (1), G542X/R1162X (1), R334W/R334W (2). Twenty five other non-carrier samples were used for validating this method. Genomic DNA (gDNA) was extracted by the salt precipitation method. The quality and concentration of gDNA was photometrically determined. All samples were kindly provided by the National Center of Medical Genetics (CNGM, Cuba).

We designed allele-specific primers to selectively amplify the wild-type and mutant alleles in parallel reactions. The selection of appropriate primers is an important factor for performing the multiplex PCR in which the specific amplification of a target requires that primers do not have matches to other targets. The same set of primers were used for detecting the mutations I507del and F508del. The simultaneous detection of both mutations allowed to reduce the number of primers in the multiplex PCR. Additionally, the same common primer was employed to detect mutations 1717-1G > A, G542X, G551D and R553X. All analyses were performed in a reaction volume of 23 μL in capped tube strips by using the SLAN 96P real-time PCR system (Shanghai Honshi Medical Technology Co., Ltd; China). Each reaction mixture contained 1× Absolute qPCR SYBR Green Mix (Thermo Scientific) and 23 primers flanking 13 targets (Table 1). During the optimization steps, primer concentrations were set to 0.1 μM, the volume was completed to 20 μL with DNase-free water and finally 3 μL of template gDNA were added. The thermal PCR profile was: 15 min at 95 °C for Thermo Start DNA Polymerase activation, followed by 27 amplification cycles (95 °C for 25 s, 59 °C for 20 s and 72 °C for 25 s). The ΔCt value of every sample was determined as the modular variation among the allele specific reactions (ΔCt = Ct mutant –Ct wild-type). Every sample with a Ct value in the mutant PCR and ΔCt < 7 was considered a carrier sample.

**Table 1:** Primer sets used for detecting CFTR gene mutations. The same set of primers was used for detecting mutations I507del and F508del. Primers flank 13 targets and allow the detection of 14 CFTR mutations

CFTR Mutation	Sequence (5′–3′)	Description	Amplicon length (bp)
R334W	TTTGTTTATTGCTCCAAGAGAGTCATACCA	antisense common primer	140
	CCTATGCACTAATCAAAGGAATCATCCTGC	sense wild type	
	CCTATGCACTAATCAAAGGAATCATCCTGT	sense mutant	
I507del/F508del	GGGTAGTGTGAAGGGTTCATATGCATAAT	antisense common primer	146
	GCCTGGCACCATTAAAGAAAATATCATTG	sense mutant	
	GCCTGGCACCATTAAAGAAAATATCATCT	sense wild type	
1717-1G > A	TAAAATTTCAGCAATGTTGTTTTTGACC	sense common primer	221
	TGTCTTTCTCTGCAAACTTGGAGATGTTC	antisense wild type	
	TGTCTTTCTCTGCAAACTTGGAGATGTTT	antisense mutant	
G542X	TAAAATTTCAGCAATGTTGTTTTTGACC	sense common primer	257
	ACTCAGTGTGATTCCACCTTCTAC	antisense wild type	
	CACTCAGTGTGATTCCACCTTCTCA	antisense mutant	
G551D	TAAAATTTCAGCAATGTTGTTTTTGACC	sense common primer	285
	GCTAAAGAAATTCTTGCTCGTTGCC	antisense wild type	
	AGCTAAAGAAATTCTTGCTCGTTGCT	antisense mutant	
R553X	TAAAATTTCAGCAATGTTGTTTTTGACC	sense common primer	291
	CACCTTGCTAAAGAAATTCTTGCTAG	antisense wild type	
	CACCTTGCTAAAGAAATTCTTGCTAA	antisense mutant	
1811 + 1,6KbA > G	TAAATTGGCTTTAAAAATTTCTTAATTG	sense common primer	135
	CAGGTGTGATTGATAGTAACCTTACTTCT	antisense wild type	
	CAGGTGTGATTGATAGTAACCTTACTTCC	antisense mutant	
2183AA > G	CAGCCAGACTTTAGCTCAAAACTCATGGG	sense common primer	170
	AACTCTCCAGTCTGTTTAAAAGATTATT	antisense wild type	
	AACTCTCCAGTCTGTTTAAAAGATTAC	antisense mutant	
3120 + 1G > A	CCTCTTACCATATTTGACTTCATCCACG	sense wild type	197
	CCTCTTACCATATTTGACTTCATCCACA	sense mutant	
	AATTTACTAAACTTATGTCTATTTTGAAGGC	antisense common primer	
3272-26A > G	CATATCTATTCAAAGAATGGCACCAGTGT	sense common primer	165
	TGCCTGTGAAATATTTCCATAGAAAACGT	antisense wild type	
	TGCCTGTGAAATATTTCCATAGAAAACGC	antisense mutant	
R1162X	TTTTGCTGTGAGATCTTTGACAGTCATTT	antisense common primer	200
	TATTTTTATTTCAGATGCGATCTGTGAGTC	sense wild type	
	TATTTTTATTTCAGATGCGATCTGTGAGTT	sense mutant	
W1282X	CCCATCACTTTTACCTTATAGGTGGGCCTC	sense common primer	178
	CCTGTGGTATCACTCCAAAGGCTTTCCAC	antisense wild type	
	CCTGTGGTATCACTCCAAAGGCTTTCCAT	antisense mutant	
N1303K	GAGAGAACTTGATGGTAAGTACATGGGTGTTTC	sense common primer	206
	GATCACTCCACTGTTCATAGGGATCCAAG	antisense wild type	
	GATCACTCCACTGTTCATAGGGATCCAAC	antisense mutant	

The mutant PCR products were melted by increasing the temperature from 70 to 90 °C at a programmed rate of 0.01 °C/s. Melting curves were analyzed with the aid of the commercial SLAN 96-P software, version 8.2.2 (Shanghai Honshi Medical Technology Co., Ltd; China).

## Results

**Fig. 1:**
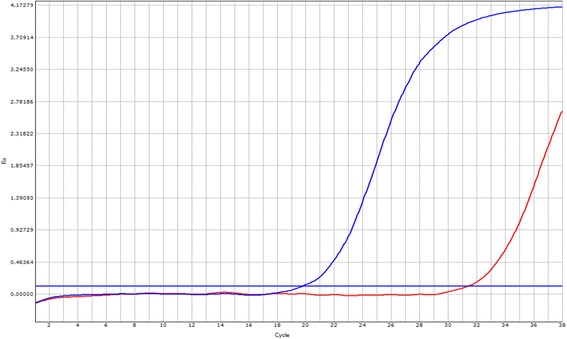
Allele-specific PCRs evaluating the trio of primers designed for detecting each mutation. Wild type curve (*blue*) and mutant unspecific amplification (*red*). All primers were tested using a non-carrier sample

In order to test the primer’s efficiency and to avoid unspecific amplification, the trio of primers designed for a mutation were tested in two simple PCRs (mutant and wild-type) (Fig. 1). Primers were tested using a non-carrier sample. For performing the multiplex PCR we only choose the trio of primers with a ΔCt greater than 7 cycles. The set of primers selected is shown in Table 1.

The PCR was set at 27 cycles, the point at which the fluorescence of the non-carrier samples in the mutant PCR increases. After PCR, the threshold was set manually over the level of a non-carrier sample (control) to avoid unspecific amplification. For detecting the presence of mutations in every sample, the mutant curve was compared with the wild-type determining the ΔCt value.

**Fig. 2:**
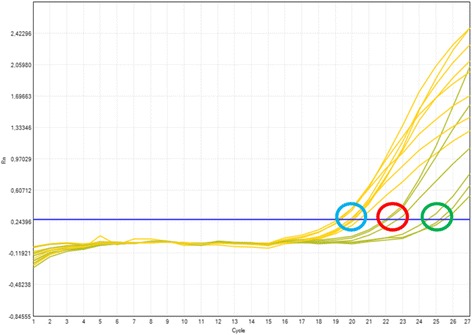
Behavior of carrier samples in the allele specific multiplex-PCR scanning 14 CFTR gene mutations. Wild type (*yellow*) and mutant (*green*) curves of samples carrying one mutation (*green* circle) or two mutations (*red* circle). The *blue* circle points out the Ct zone of the wild type PCR

**Fig. 3:**
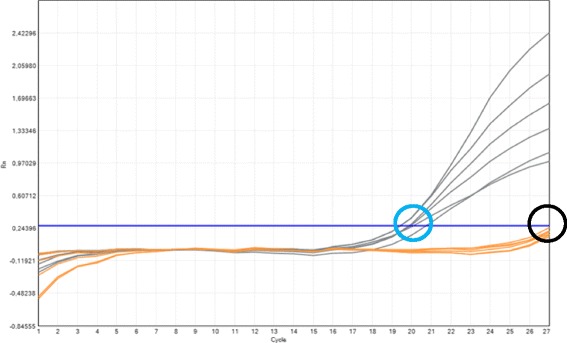
Behavior of non-carrier samples in the allele specific multiplex-PCR scanning 14 CFTR gene mutations. Wild type (*gray*) and mutant (*orange*) curves. The *blue* circle points out the Ct zone of the wild type PCR, the *black* circle points out the unspecific amplification of mutant PCR

The behavior of carrier and non-carrier samples in the allele specific multiplex-PCR scanning 14 CFTR gene mutations is shown in Figs. 2 and 3 respectively. As shown in Fig. 2, a sample which carries at least one of the 14 selected mutations displays a Ct value before cycle 26 and the ΔCt < 7. Non-carrier samples do not display a mutant Ct value (Fig. 3). The number of affected alleles (zygosity) was also distinguishable. All samples carrying one allele have a 3 < ΔCt < 7 while samples carrying two alleles in *trans* have a ΔCt < 3 (Fig. 2). Particularly, samples with F508del homozygous genotype have ΔCt < 1. Samples with the Ct of the wild-type curve over cycle 21 and ΔCt < 7 were not in the appropriate range of concentration. They were retested at higher concentrations (>50 ng of total DNA).

**Fig. 4:**
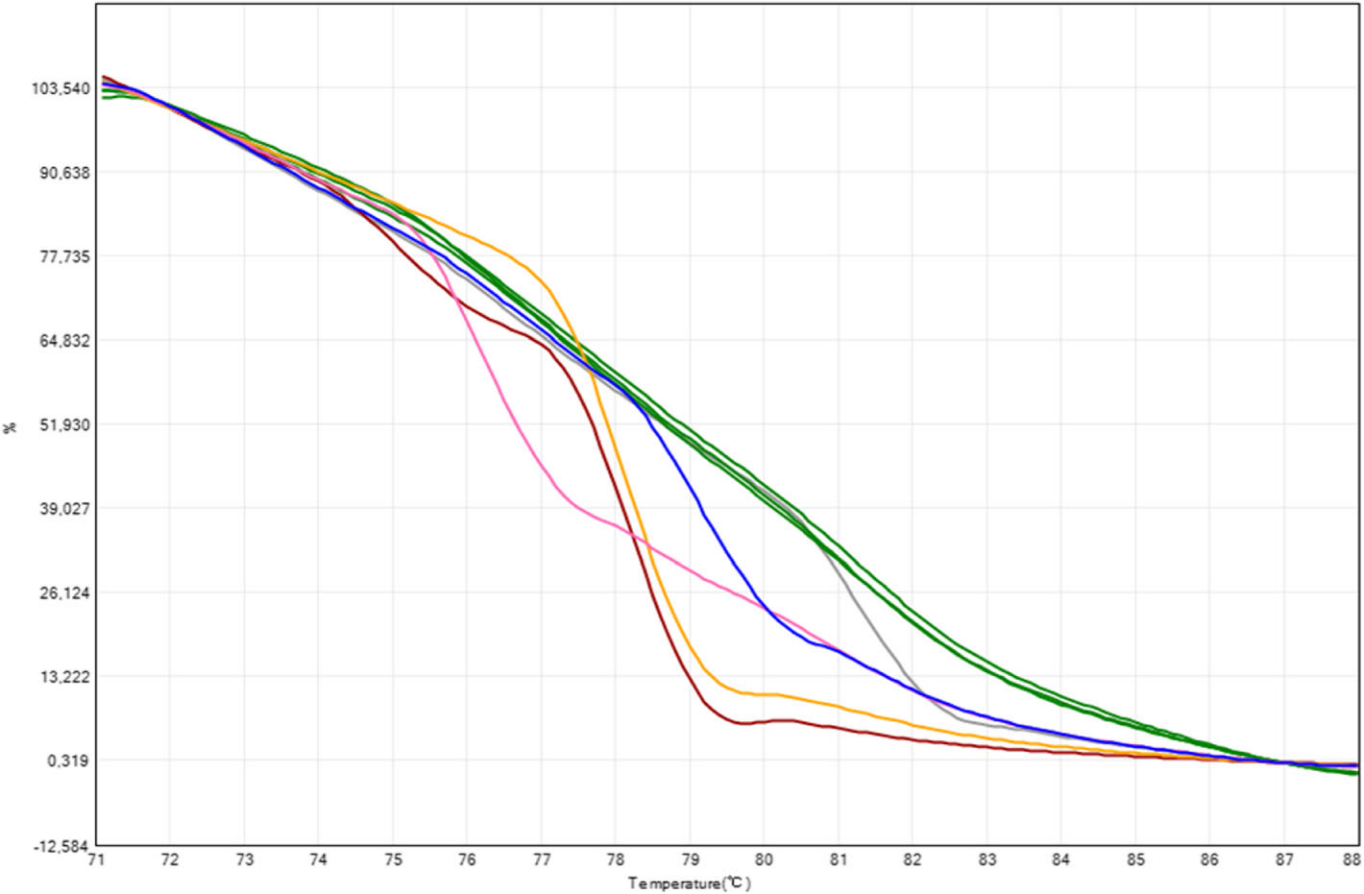
Determination of the genotype of the sample in the mutant allele specific multiplex PCR. Melting curves obtained at a ramp rate of 0.01 °C/s. Each color represents a genotype: *green* (non-carrier), *blue* (R553X), *pink* (3120 + 1G > A), *brown* (F508/N1303K), *grey* (R334W) and *yellow* (G542X). HRMA was performed to allow the identification of the mutations when possible

**Fig. 5:**
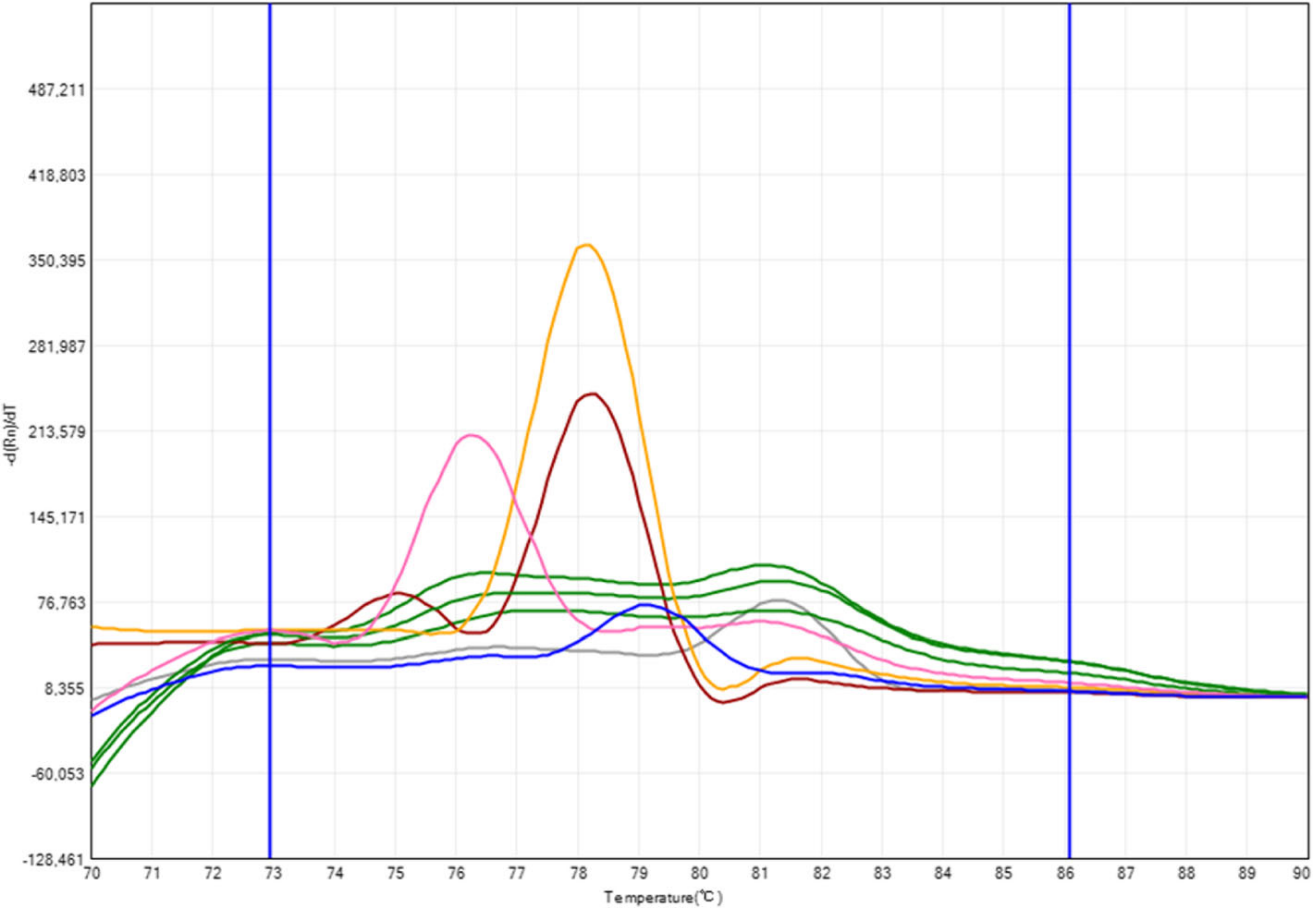
Derivative melting curve showing the melting peaks of mutant PCR products. The peaks represent the melting temperature of the specific amplified target. HRMA was performed to allow the identification of the mutations when possible

The HRMA of mutant PCR products confirmed the presence of mutations (Fig. 4). The derivative melting curves indicated the melting temperature of the most amplified target in the multiplex PCR (Fig. 5); this target can only be amplified when the mutation is present. A melting temperature profile was obtained for every amplicon. Samples carrying two affected alleles generating amplicons with close temperature profiles could not be correctly resolved using HRMA.

All the CFTR gene mutations were detected in every carrier sample. HRMA efficiently confirmed mutations according to the melting temperature of the amplicon: R334W (81.30 °C), I507del (78.42 °C), F508del (78.21 °C), 1717-1G > A (77.14 °C), G542X (78.08 °C), R553X (79.11), 2183AA > G (78.80 °C), 3120 + 1G > A (76.26 °C), R1162X (81.80 °C) and N1303K (75.04 °C).

## Discussion

We have developed a simple method for scanning 14 selected CF-causing mutations with high frequency in Latin America. Related studies have been recently reported [10, 11, 16]. We only included the scanning of 14 highly frequently CFTR mutations because of the primers limited availability at the time of this study, but some other mutations could potentially be detected by using this method. By determining the ΔCt value between two allele-specific PCRs, we detected the presence of at least one CFTR mutation in every carrier sample. This method will allow us to confirm or discard a sample in a high-throughput scanning of CFTR mutations.

Studies such as this in cystic fibrosis are scarce and very limited, including just a few mutations in the analysis [16, 17]. Our study highlights the development of a multiplex allele-specific real-time PCR assay for the detection of mutations in the CFTR gene. This offers a significant savings of time and labor compared with the singleplex PCR analysis and provides more qualitative information. Furthermore, the ability to carry out the detection of 13 targets using the same channel is another advantage that reduce the complexity of the necessary equipment. Added to all these benefits the subsequent use of HRMA allows to discriminate the PCR products according to their melting temperature (Tm). The combination of allele-specific PCR and HRMA enables to obtain a greater reliability in the results of the samples being analyzed [18, 19].

We did not have any sample carrying the genotypes G551D, 1811 + 1,6KbA > G, 3272-26A > G or W1282X, however, we included those primers in the multiplex PCR due to the high frequency of these mutations in Latin-American countries and their probable presence in the Cuban population. Their inclusion in this scanning technique validates the selectivity of all primers. This strategy could be the most cost-efficiently way to detect these unreported genotypes.

Starting gDNA concentration is a critical factor that could change the interpretation of the PCR curves. All samples tested using this method should have amounts of gDNA over 50 ng. Samples below this value will have the Ct of the wild-type curve two or three cycles later and lower ΔCt, producing misleading results. Although the required DNA quantity (>50 ng) is an important parameter to consider for avoiding late amplification, many DNA extraction methods can easily yield this amount.

A Ct value in the mutant PCR indicates the presence of at least one mutation while the ΔCt value indicates the number of affected alleles (1 or 2). This method simplifies the detection of a carrier sample facilitating the analysis of multiple mutations. The risk of contamination is also reduced because the amplified products never leave the reaction tube.

The HRMA of mutant PCR products confirmed the presence of mutations. The derivative melting curves indicate the melting temperature of the most amplified target in the multiplex PCR. HRMA is a useful technique for scanning exons, but in our study; it was performed to complement the prediction of PCRs, allowing the identification of mutations when possible.

Although current high-throughput sequencing technologies emerge now for screening and detection of somatic mutations, their cost remains high enough to be considered for routinary use in developing countries. Other findings call into question the sensitivity of sequencing methods detecting defined somatic mutations on clinical samples [20, 21]. Taking into account the aforementioned advantages including necessary equipment and reagents availability, we justify and recommend the use of the proposed approach prior to the use of non-specific scanning or sequencing methods in the CF diagnostic algorithm.

## Conclusions

The results published in this work could be the start of a new way to detect CFTR mutations in the Cuban newborn and carrier-screening programs for CF. This approach provides some additional advantages: the cost of mutation screening of the CFTR gene and the time for completing the analysis were reduced. Every sample with suspicion of having mutations could then be reconfirmed and genotyped by using HRMA which increases the reliability of the result. This method could be reproduced in any laboratory with a calibrated real time PCR system. This strategy could also be applicable to any gene, particularly large genes with heterogeneous mutation spectrums.

## Abbreviations

ARMS: Amplification Refractory Mutation System
CF: Cystic fibrosis
CFTR: Cystic Fibrosis Transmembrane Conductance Regulator
CNGM: National Center of Medical Genetics
Ct: Cycle threshold
DGGE: Denaturing gradient gel electrophoresis
gDNA: Genomic DNA
HRMA: High Resolution Melting Analysis
PCR: Polymerase Chain Reaction
wt: Wild-type
